# Hybrid model: a promising type of public procurement in the healthcare sector of the European Union

**DOI:** 10.3389/fpubh.2024.1359155

**Published:** 2024-02-15

**Authors:** Nikolaos Geropoulos, Polychronis Voultsos, Miltiadis Geropoulos, Fani Tsolaki, Georgios Tagarakis

**Affiliations:** ^1^Postgraduate Master's Programme: Health and Social Care Services Management, Faculty of Health Sciences, School of Medicine, Aristotle University of Thessaloniki, Thessaloniki, Greece; ^2^Laboratory of Forensic Medicine & Toxicology (Division: Medical Law and Ethics), Faculty of Health Sciences, School of Medicine, Aristotle University of Thessaloniki, Thessaloniki, Greece; ^3^Master of Science in Business Administration Programme, Faculty of Economics and Business, Katholieke Universiteit Leuven, Brussels, Belgium; ^4^Postgraduate Master's Programme: Health and Social Care Services Management, Faculty of Health Sciences, School of Medicine, Aristotle University of Thessaloniki, Thessaloniki, Greece; ^5^Department of Cardiothoracic Surgery, Faculty of Health Sciences, School of Medicine, Aristotle University of Thessaloniki, Thessaloniki, Greece

**Keywords:** public procurement, health system, medical supplies, centralization, hybrid model, European Union

## Abstract

The management of health supplies in public hospitals has been a major concern of national and European institutions over time, often being a field of reforms and regulatory interventions. Health procurement systems constitute complex decision-making and supply chain management mechanisms of public hospitals, involving suppliers, health providers, administrators and political bodies. Due to this complexity, the first important decision to be taken when designing a procurement system, concerns the degree of centralization, namely to what extent the decision-making power on the healthcare procurement (what, how and when) will be transferred either to a central public authority established for this purpose, or to the competent local authorities. In this perspective, we attempt to analyse the types of public procurement in the healthcare sector of the European Union, in terms of degree of centralization. Employing a narrative approach that summarizes recent interdisciplinary literature, this perspective finds that the healthcare procurement systems of the EU Member States, based on the degree of centralization, are categorized into three types of organizational structures: Centralized, Decentralized and Hybrid procurement. Each structure offers advantages and disadvantages for health systems. According to this perspective, a combination of centralized and decentralized purchases of medical supplies represents a promising hybrid model of healthcare procurement organization by bringing the benefits of two methods together.

## 1 Introduction

The health systems worldwide are faced with the increasing cost of medical care, the limitation of available resources and the failure to meet the legitimate and reasonable expectations of patients-users in relation to the quality of services provided. Addressing costs, supply shortages and treatment disruption in complex settings can be critical to strengthening health systems (1). Nowadays, the users of health services, worldwide, seek equal access to increasingly better quality healthcare and expect the use of medical equipment and modern medical diagnostic tools to them that incorporate the latest technology (2). Implementing the equal access obligation (3) and ensuring the provision of high-quality public health services, requires a strategic approach to sourcing, which depends to a large extent on modern and efficient public procurement procedures (4).

For all the reasons above, the management of healthcare supplies in public hospitals has been a serious concern for the Member-States (M-S) of the European Union (EU). Changes in healthcare procurement include centralizing or decentralizing procurement, improving information systems to track and update data, improving infrastructure and processes along the supply chain (1). Therefore, the solution to successfully address these problems in the healthcare sector lies in the rational management of public hospitals' supplies or otherwise in the effective management of their supply chain (5).

In this perspective, we approach the issue of healthcare procurement in the EU M-S in light of the degree of centralization and we attempt to compare the types of organizational structures, in order to draw useful conclusions about their advantages and disadvantages. The aim of this discussion is to inform policymakers on promising cost containment policies as well as best practices to improve the efficiency, transparency, and competition of their healthcare procurement systems.

## 2 The importance of degree of centralization in public healthcare procurement

The total volume of EU healthcare expenditure in 2020 amounted to 10.91% of its total gross domestic product (GDP) (6, 7). Hospitals accounted for the highest proportion (37.4%) of healthcare expenditure in 2020 in the EU (8). Medical goods were the second largest function in the EU in 2020, with an 18.2% share of current healthcare expenditure (9). An analysis of current healthcare expenditure in the EU M-S is shown in Table 1.

**Table 1:** Healthcare expenditure in the European Union in 2020.

**EU M-S**	**% of GDP**	**Hospitals (% of healthcare expenditure)**	**Medical goods (% of healthcare expenditure)**
Austria	11.5	38.7	16.2
Belgium	11.1	41.8	13.1
Bulgaria	8.5	39.8	34.1
Croatia	7.8	46.8	23.2
Cyprus	8.1	45.6	14.2
Czechia	9.2	45.5	17.3
Denmark	10.5	46.3	10.5
Estonia	7.8	44.2	19.8
Finland	9.6	37.7	14.2
France	12.2	38.9	19.4
Germany	12.8	28.8	18.2
Greece	9.5	43.8	32.5
Hungary	7.3	40.3	28.1
Ireland	7.1	37.3	12.6
Italy	9.6	44.8	20.8
Latvia	7.5	34.6	26.2
Lithuania	7.5	33.8	26.9
Luxembourg	5.8	33.1	12.8
Malta	9.2	40	24
Netherlands	11.1	33.3	10.6
Poland	6.5	40.4	21.7
Portugal	10.6	43.2	19.8
Romania	6.3	48	25.9
Slovakia	7.2	34.7	31.8
Slovenia	9.5	39.1	21.1
Spain	10.7	46.4	21.1
Sweden	11.4	39.7	12.2
EU	10.9	37.4	18.2

Source: Developed by authors based on the data of the Eurostat Statistics (online data codes: hlth_sha11_hf and nama_10_gdp).

Due to the significant volume of healthcare expenditure, the main objective of M-S is to optimize their supplies so that health systems offer maximum efficiency in relation to the financial, material and human resources used. Their initiatives include centralized or decentralized supplies, the enhancement of information systems for monitoring and updating data and the improvement of infrastructures and processes along the supply chain (10). On public procurement in particular, the European Parliament and the Council adopted in 2014 a new package of measures, which includes Directive 2014/24/EU (11).

The efficiency and budget control at a public healthcare provider can vary significantly depending on how their procurement is organized and managed. One way to increase the efficiency and effectiveness of public procurement is to choose either between a centralized system, where a central body is responsible for managing all purchasing and procurement activities (selecting suppliers, negotiating prices and terms, making a purchase decision) for final recipients (i.e., local units), who are simply asked to send their requests to it, or a decentralized system; where local units procure themselves (12).

Therefore, the first important decision to be taken in designing the procurement system of a complex organization, such as the health system, concerns its degree of centralization (13). This decision falls under the more general issue of award, an issue that has been widely explored, although not very extensively in public procurement. The issue of centralization vs. decentralization of procurement, due to both the need to control costs and the rationalization of procedures, attracts the interest of researchers, professionals and public administration executives from various angles and is becoming increasingly important for many organizations (14).

## 3 The three types of healthcare procurement systems based on the degree of centralization

With the aforementioned research approach, we will examine the various models of healthcare procurement based on the degree of centralization and we will review the arguments for and against centralized and decentralized procurement operations. Then we will try to consider the possible impact of such systems in the healthcare sector. In this perspective, the procurement systems based on the degree of centralization and the extent of the powers of contracting authorities are classified into three main types (15).

### 3.1 Centralized procurement

**Figure 1:**
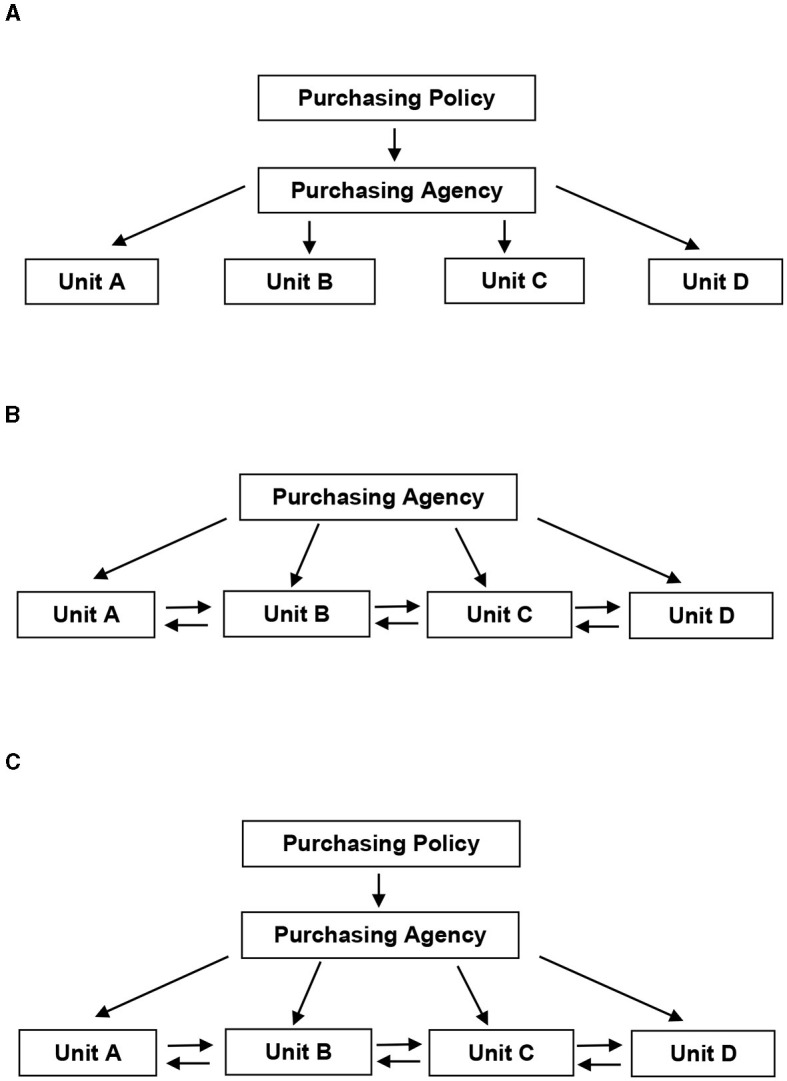
Schematic diagrams of decision-making authority. **(A)** Centralized model. **(B)** Decentralized model. **(C)** Hybrid model. Source: McCue and Pitzer (16).

A procurement system is fully centralized when all relevant decisions (what, how and when) on the purchasing of products, whether through tendering procedures or negotiations, are taken by a central purchasing body set up for this purpose, as shown in Figure 1A (16).

According to Directive 2014/24/EU “*Central purchasing body means a contracting authority providing centralized purchasing activities and, possibly, ancillary purchasing activities”* (Directive 2014/24/EU of the European Parliament and of the Council of 26 February 2014 on Public Procurement and Repealing Directive 2004/18/EC Text with EEA Relevance, 2014). At this point, we have to highlight that Directive 2014/24/EU pursuant to recital no. 59 et seq., establishes the separation between aggregation and centralization. The distinction between centralization and aggregation, is that the “centralization” is related to the preparation of a Public Procurement Framework Agreement, whereas the “aggregation” is linked to the concept of quantity or the batch size of procedures. This Directive also emphasizes Dynamic Acquisition Systems, having adjusted the rules applicable to this contracting instrument so that entities adjudicators can take advantage of this instrument. Pursuant to article 37 of the Directive referred to in the previous paragraph, thus becomes one of the possible instruments for use in the scope of centralized purchasing. Furthermore, the terms of the contract for the products under supply are the same in all local public bodies. Centralized procurement and competitive tendering can achieve cost savings across multiple sectors by reaping economies of scale and improved purchasing power. Moreover, the benefits are not only lower prices and savings but also they include improved transparency and governance such as enhanced equity (17). According to Coe centralization of procurement procedures is necessary, both to prevent waste and inefficiency, and to establish control systems (18). It also helps to reduce the situations of exceptional purchases, which usually occur when staff are not sufficiently trained at professional level in purchasing methods.

Undoubtedly, the centralization of public procurement is becoming established throughout the EU. After a period of skepticism, where central procurement was seen as a factor of monopsony and reduced competition, central purchasing bodies, as aggregators, are now managing increasing shares of public procurement. In this spirit, the European Commission's Communication explicitly recognizes the possibility of setting up central purchasing bodies with a general mandate at national level, targeting specific sectors, including health. Given the large volume of public procurement achieved by central purchasing bodies, they can be used to leverage strategic procurement, such as targeting public procurement. They could also play an important role in standardization of public procurement procedures. Central purchasing bodies, by virtue of their specialized knowledge and expertise in purchasing issues, may provide support and advisory services to other contracting authorities and thus contribute indirectly to improving the professional character of public administrations (4).

For the healthcare sector in particular, centralization of procurement is not a new idea. Research by Board on Global Health (BGH) illustrates the benefits of a centralized international malaria drug procurement system (19). Similarly, the Supply Division of United Nations Children's Fund (UNICEF) in Copenhagen has many years of experience in the fully centralized vaccine market under the umbrella of the public-private health partnership Global Alliance for Vaccines and Immunization (GAVI), established in 2000 with aim to increase equitable access to immunization in lower income countries (20). Similarly, the World Health Organization (WHO) proposed similar initiatives at international level to reduce costs in tackling malaria, tuberculosis and HIV/AIDS (21).

In summary, the main arguments in favor of the centralized procurement systems are the following (22):

(a) Significant price reductions.
(b) Better goods and services at lower cost.
(c) Increased purchasing power.
(d) Need for technical standardization (e.g., in the field of information systems and software applications).
(e) Definition and inclusion in the terms of environmental standards for public procurement.
(f) No cost benefits, including greater attention to contract management and better problem solving (e.g., defective products, substandard services, inadequate after-sales services).
(g) Lower staff training costs because they are numerically less and centrally located.
(h) Easier management of staff performance.
(i) Encourage the issuance of transparency regulations, such as effective recording and reporting of procurement and transaction contracts, effective management controls.

On the other hand, the opponents of centralization contend that the expenses related to time and effort necessary to establish and maintain cooperative interactions, such as transaction costs (e.g., information, negotiation, and enforcement costs), could surpass the potential advantages of centralization. Moreover, as centralization takes place, there is a growing concern that it might gradually exclude peripheral actors from participating in governance. This exclusion can potentially limit the ability of central governments to effectively address disputes or develop policies that require local input (23).

### 3.2 Decentralized procurement

Fully decentralized is the procurement system in which the power of decision making on the purchasing of products (what, how and when) has been transferred to the relevant local administrations, as shown in Figure 1B (16).

The decentralization of the health system can be implemented in various forms and to different degrees, depending on the existing policy, the public administrative structure and the organization of the health system of each country. The most effective programs that improve supply chain and procurement processes address the root causes of system inefficiencies and may provide specific interventions (24).

The logic of decentralization is based on the fact that smaller organizations, properly organized and managed, are inherently more flexible and responsible than larger organizations. Another rationale is that by placing procurement management closer to end-user needs, it is likely to be more cost-effective and able to promote the growth of the private sector, including small and medium-sized enterprises (22). Even the German sociologist Max Weber, who first formulated the basic features of the bureaucratic model and who himself reluctantly concluded that bureaucracy was inevitable in human organization, considered that the only alternative to bureaucracy is a return to small-scale organization (25). Given the strength of this view, it is not surprising that from time to time, and especially since World War II, in an effort to restructure the health sector in various European countries, many national and regional policymakers have introduced decentralization strategies.

Moreover, another important reason for the attractiveness of the idea of decentralization of systems is its adaptability, which allows it to fit simultaneously into the many different national and local health policies. Thus, the decentralized bodies in the health sector can either be public institutions financed by taxes, or non-profit private entities such as sickness funds in countries that have the social health insurance system or even private companies listed on the stock exchange, for example insurance companies.

In summary, the main arguments of the proponents of the idea of decentralization of public procurement are the following (22):

(a) Reduced incentives for corruption through protectionism or large-scale favoritism.
(b) A better responsiveness of procured goods to the detailed end-user requirements.
(c) Reduced margin of error in contrast to those of large purchases resulting in wasteful and excessive spending.
(d) Less bureaucracy, due to shorter time frames and fewer required forms (applications, supporting documents) for both buyers and suppliers.
(e) Greater opportunities for successful competition for small and medium-sized enterprises.
(f) Opportunities for local buying authorities to purchase at lower prices the locally manufactured products.
(g) Increased scope for work responsibility by employees and development of a spirit of “service.”

However, such a wide variety of decentralized institutional forms raises a number of questions about the key features of decentralization and the ability of smaller decentralized units to respond effectively to the demands of modern healthcare, such as the provision of integrated care to patients with chronic diseases or older adult patients. Questions also arise regarding both the managerial ability of decentralized units to organize and their financial ability to procure key new technologies for their clinical and information systems (26). Collusion and corruption at local level can be another thorny issue, as decentralization can result in a loss of public oversight and quality assurance of products in procurements and the supply chain. But also from an economic aspect, a decentralized approach may promote the development of the private sector and SMEs as mentioned above, but on the other hand may sacrifice the potential advantages of centralized purchasing and the achievement of economies of scale.

In recent years, the above concerns have created a reversal of the trend of decentralization of health systems in some countries to more centralized systems. In particular, the Nordic countries of the EU, such as Sweden, Denmark and Finland but also Central European countries such as Poland and Slovakia which had adopted strategies of decentralization in the health sector, are beginning to recentralize basic functions in their health systems (22).

### 3.3 Hybrid procurement

Between the decentralized and centralized procurement system, there is also an intermediate system, the hybrid system, in which the central authority and the competent local administrations share decision-making power, as shown in Figure 1C (16).

A quite common example of a hybrid procurement system, is the framework agreement that central purchasing body may conclude on behalf of their regional public administrations (15). According to Directive 2014/24/EU“* framework agreement means an agreement between one or more contracting authorities and one or more economic operators, the purpose of which is to establish the terms governing contracts to be awarded during a given period, in particular with regard to price and, where appropriate, the quantity envisaged”* (Directive 2014/24/EU of the European Parliament and of the Council of 26 February 2014 on Public Procurement and Repealing Directive 2004/18/EC Text with EEA Relevance, 2014).

A framework agreement may be concluded:

(a) By a contracting authority to meet its own needs (e.g., the Ministry of Health concludes a framework agreement with three suppliers for the supply of stationery for its departments).
(b) By several contracting authorities or by a contracting authority operating on behalf of several contracting authorities (e.g., the Regional Health Agency concludes a framework agreement with a contractor for the supply of needles to hospitals in its jurisdiction).
(c) By a central purchasing body, acting on behalf of other contracting authorities (e.g., the National Central Health Procurement Authority concludes a framework agreement for all hospitals in the country, with five suppliers for the supply of specific medical equipment).

At this point, it should be noted that the framework agreement is not a new type of public contract, but a contractor selection method, which is not legally binding, unlike individual contracts concluded on the basis of such agreements (27). Therefore, in such a mild centralized arrangement as hybrid, the individual implementing contracts, concluded on the basis of the framework agreement make available to all regional public administrations, various goods to be procured for a given period of time at a specific (often renegotiated) price. Public administrations have the discretion to decide whether to conclude an individual implementing contract on the basis of the framework agreement (recommended), unless the required goods are not available or local suppliers are able to provide similar products in a better price and quality.

For the healthcare sector in particular, the hybrid procurement system can benefit the performance of the healthcare system with some decentralized functions. For example, it can benefit the financing and the programming-drafting of budget, since these functions are likely to require greater flexibility so that they respond to local information. It can also benefit other more centralized functions, such as inventory control, warehousing, product transportation, logistics management information systems, since these functions can benefit from supervision, storage capacity, etc. In addition, the hybrid procurement system can serve national and regional programmes, with central government playing an essential role in the procurement, storage and distribution of selected public health products, such as vaccines (24).

## 4 Discussion on healthcare procurement systems in the European Union countries

In light of the degree of centralization, we analyse the healthcare procurement system of the European Union countries. Performing a theoretical literature research in combination with the collection, recording, processing and comparison of EU healthcare procurement policies, we attempt to ascertain which type of healthcare procurement is implemented by each EU country and whether or not a central purchasing body (CPB) has been established. The theoretical literature research conducted through valid search engines (PUBMED, Google Scholar) includes scientific articles and electronic repositories, relevant and appropriate publications from primary and secondary sources, bibliography, press articles, conference summaries, legislation and jurisprudence.

The results obtained by the analysis of the healthcare procurement systems of the EU countries, regarding the type of health procurement system based on the degree of centralization as well as the operation of CPBs are as follows (see Table 2):

(a) The EU countries implementing the “centralized” healthcare procurement system are Austria (28–30), Bulgaria (29, 31–33), Cyprus (17, 34, 35), Denmark (17, 36–39), Ireland (29, 40–42), Latvia (29, 43–46), Lithuania (29, 47, 48), Luxembourg (29, 49–51), Malta (29, 52, 53), Portugal (17, 29, 54–58), Romania (29, 59–61), Slovenia (29, 62, 63) and Spain (64–67). There is no tendency toward decentralization in the above countries. Almost all EU countries operating a centralized health procurement system (except Luxembourg and Slovenia) have established central purchasing bodies which are contracting authorities providing centralized purchasing activities.
(b) The EU countries implementing the “decentralized” healthcare procurement system are Belgium (29, 68–72), Czechia (29, 73–75), Estonia (76–78), Finland (29, 38, 43, 79), France (29, 80–82), Germany (29, 83–85), Poland (29, 74, 86), Slovakia (29, 87, 88) and Sweden (29, 89, 90). The vast majority of these countries have in recent years implemented policies of gradual centralization of their procurement in order to achieve economies of scale, save resources, increase transparency of public spending and fight corruption.
(c) The EU countries implementing the “hybrid” healthcare procurement system are Croatia (10, 91–93), Greece (94–97), Italy (12, 29, 38, 98–101) and Hungary (29, 74, 102, 103)).

**Table 2:** Types of healthcare procurement in EU member states.

**EU M-S**	**Type**	**Change tendency**	**Centralized purchasing body**
Austria	Centralized		Yes, BBG
Belgium	Decentralized	Centralized	Yes, Mercur
Bulgaria	Centralized		Yes, CCPP
Croatia	Hybrid		Yes, CPO
Cyprus	Centralized		Yes PSD
Czechia	Decentralized	Centralized	No
Denmark	Centralized		Yes, AMGROS
Estonia	Decentralized	Centralized	No
Finland	Decentralized	Centralized	Hansel Oy
France	Decentralized	Centralized	Yes, RESAH
Germany	Decentralized	Centralized	Yes, multiple
Greece	Hybrid		Yes, NCHPA
Hungary	Hybrid		Yes, OKFO
Ireland	Centralized	Centralized	Yes, OGP
Italy	Hybrid		Yes, Consip
Latvia	Centralized		Yes, procurement division of NHS
Lithuania	Centralized		Yes, CPO
Luxembourg	Centralized		No
Malta	Centralized		Yes, CPSU
Netherlands	Decentralized		No
Poland	Decentralized		No
Portugal	Centralized		Yes, SPMS
Romania	Centralized		Yes, ONAC
Slovakia	Decentralized	Centralized	No
Slovenia	Centralized		No
Spain	Centralized		Yes, multiple
Sweden	Decentralized	Centralized	No

Source: Developed by authors based on the data of the EU Country profiles of the European Observatory on Health Systems and Policies (HiT series) and the Public procurement – Study on administrative capacity in the EU.

Procurement is seldom exclusively centralized or decentralized in its entirety (104, 105). In order to classify the healthcare procurement on the basis of centralization, we must first identify the range of capabilities offered within a gradual scale, ranging from minimal coordination between healthcare providers to full bulk purchasing. The choice between a fully centralized and a fully decentralized procurement system comprises some different intermediate forms of operation (106). For example, in the case of Portugal, which applies a mild centralized type of procurement, whose characteristics are quite similar to the hybrid one (107), we have identified four levels of centralization as below:

(a) Centralized

The Centralized model concerns only the development of Framework Agreements, with activity being directed toward issues of market assessment and establishment of technical requirements. This activity is carried out in conjunction with experts from other institutions, whose objective is to regulate the purchase, through the standardization of the technical requirements of the product or service to be purchased. Through this process, the market operators who are able to meet the terms of the agreement are selected and committed to supply their products with the required technical requirements and the agreed prices during the validity of the framework agreement. Healthcare providers develop their respective acquisition procedures under the Framework Agreements.

(b) Centralized aggregate

In the Centralized Aggregate model, in accordance with the Framework Agreements, acquisition processes are developed for a group of institutions, acting on their behalf and as a purchasing center.

(c) Aggregate

In the Aggregate model, a competent body acts as a purchasing center, but the contracting instrument used is not the Framework Agreement, but other public contracting instruments such as public tenders.

(d) Free

The Free model is used by institutions to acquire goods or services that are not included in the other three models, with each institution developing its own acquisition processes.

Several studies have shown that centralized and hybrid procurement systems perform better with respect to decentralized systems (12, 100, 108–110). Based on numerical experimentation, it is generally found that a hybrid combination of the centralized and decentralized procurement types tends to yield the optimal results (104, 110).

## 5 Conclusions

In this perspective article, employing a narrative approach that summarizes recent interdisciplinary literature on healthcare procurement systems of the European Union countries based on the degree of centralization, we identified three types of organizational structures: Centralized, Decentralized and Hybrid procurement. We discussed the three different ways to organize the procurement function, as well as the advantages and disadvantages of each structural organization. We found that the implementation of healthcare procurement centralization or decentralization in the EU can vary in terms of its forms and levels, influenced by the political and administrative structure of a M-S and the organization of its health system. By consolidating procurement and tendering processes, centralization can generate cost savings in various settings through the advantages of scale and enhanced purchasing ability. For this reason, there has been a growing strategic trend toward centralizing purchases, especially within the EU healthcare systems. Health system performance can be improved through a hybrid procurement model, where certain functions are decentralized, such as financing and planning/budgeting and conversely, other functions, such as control, storage and logistics can be centralized. In this perspective, a combination of centralized and decentralized purchases of medical supplies could represent a promising hybrid type of healthcare procurement organization by bringing the benefits of two systems together. But, striking the appropriate equilibrium between centralization and decentralization is a highly intricate challenge. To effectively combine the potential advantages of both approaches, it is necessary to carefully establish a balance and define the relationships between local and central management of hospital supplies. Policymakers should consider the best practices and lessons learned from each country to improve the efficiency, transparency, and competition of their healthcare procurement systems.

## Data availability statement

The original contributions presented in the study are included in the article/Supplementary material, further inquiries can be directed to the corresponding author.

## Author contributions

NG: Conceptualization, Data curation, Formal analysis, Investigation, Methodology, Visualization, Writing – original draft, Writing – review & editing. PV: Formal analysis, Supervision, Validation, Writing – review & editing. MG: Data curation, Formal analysis, Writing – review & editing. FT: Formal analysis, Validation, Writing – review & editing. GT: Formal analysis, Project administration, Supervision, Validation, Writing – review & editing.
