# Supplementary material for: Hybrid model: a promising type of public procurement in the healthcare sector of the European Union
Source: Front Public Health. 2024 Feb 15;12:1359155. doi: 10.3389/fpubh.2024.1359155 (PMC10902422; doi:10.3389/fpubh.2024.1359155)
Supplement: Supplementary file 1 [file Presentation_1.pdf]

Hybrid model

A promising type of public procurement in the healthcare sector of the EU

OBJECTIVE:
THE MODELS OF PUBLIC HEALTH PROCUREMENT BASED ON THE DEGREE OF CENTRALIZATION

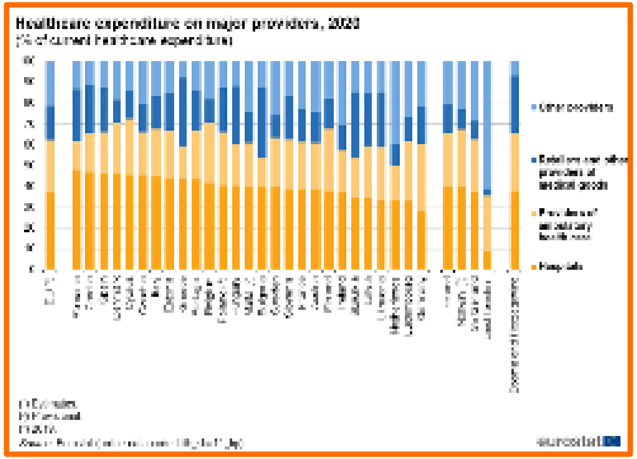

Hospitals (37,4%) & Medical goods (18,2%): The 2 highest proportions of healthcare expenditure in the EU.

RESULTS:
THREE TYPES OF PUBLIC HEALTH PROCUREMENT IN THE EU

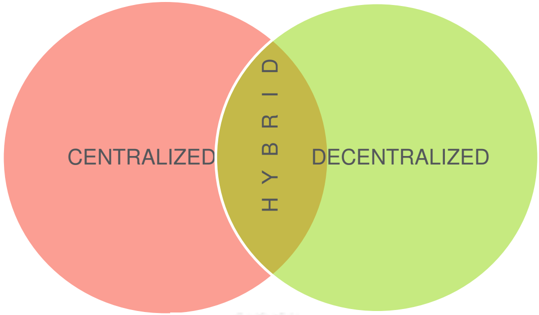

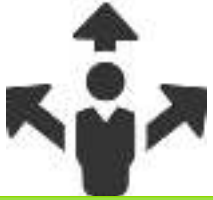

PROCUREMENT SYSTEM :
COMPLEX DECISION MAKING MECHANISM

CONCLUSION:
HYBRID PROCUREMENT-AN EFFECTIVE COMBINATION OF CENTRALIZED AND DECENTRALIZED ADVANTAGES

BENEFITS OF DECENTRALIZED FUNCTIONS	BENEFITS OF CENTRALIZED FUNCTIONS
REDUCED BUREAUCRACY	INVENTORY CONTROL
LOCAL SOURCING	STORAGE CAPACITY
FASTER PURCHASING	COST SAVINGS
FLEXIBILITY	INCREASED TRANSPARENCY

frontiers in Public Health

Authors: Geropoulos N, Voultsos P, Geropoulos M, Tsolaki F, Tagarakis G

Reference: Geropoulos N, et al. Hybrid model: A promising type of public procurement in the healthcare sector of the European Union. *Front. Public Health*, (2024), doi: 10.3389/fpubh.2024.1359155
